# Care of the Hair

**Published:** 1881-03

**Authors:** 


### CARE OF THE HAIR.

UNLESS there is disease of the scalp, or an irritation that produces dandruff, the hair requires little attention beyond the most scrupulous cleanliness.

Nature supplies the hair with a soft glossy oil, perfectly adapted to the purpose, and whose essential qualities no chemist has the skill to counterfeit.

When the scalp and the hair are encumbered by accumulations of dust, which combine with the natural oil to form a dark and offensive pigment, they must be cleansed. Men can perform this ceremony easily and quickly, since they have only to wet the head with warm water in winter and cold water in summer, and then rub with soap for a minute, and afterward apply plenty of water with the hands, while bending over a basin of water. When the soap has been thoroughly washed out, dry with towels.

Women with long hair (we take no interest in the short-haired variety) should have assistance in washing the hair. The best way is to sit in a chair with the head over a large vessel of warm water, which may be placed on another chair or on the floor. The hair should be thrown forward so as to fall over the face. This will prevent the clothing from becoming wet. The assistant should rub soap well into the hair and on the scalp, with the hands or a sponge; and then pour on a little water occasionally while rubbing, until the soap is washed away and the scalp looks clean and white. The hair should then be rubbed with dry towels until there is but little moisture left.

When dry it will have a clean and rich and glossy appearance, that no pomade or hair preparation, except soap and water, could give it. When once the hair is thus cleansed, it is an easy matter to keep it soft and beautiful. Apply cold water every morning with the hands and wipe dry with a towel. For a few days the hair may seem harsh and dry, but perseverance in this course is sure to be rewarded with a soft, glossy and healthy head of hair; and this is the reason. Nature has provided for the manufacture and secretion, as it is required, of an oil adapted to the growth and beauty of the hair; and if the parts are kept cleansed she will do her work perfectly. Thus we see that children generally have the most beautiful hair, because nature is not interfered with in her work. But men and women usually think that they can improve on nature, and they invent and apply all sorts of lotions and unguents, and comb and brush the hair until they produce irritation of the scalp, and thus obstruct the secretion of oil, and the scalp becomes coated thickly with dandruff and the hair dry and lifeless.

The scalp should seldom be touched with a fine tooth comb nor a stiff brush. Such combing and brushing produces irritation and disease, just as similar treatment would produce irritation and disease on any other part of the body. If the scalp is already diseased it should be treated with soap and warm water, as above described, until it is brought into a healthy condition, and it may be kept so by applying cold water daily, which keeps the scalp cool and healthy, the hair fine and glossy, and prevents premature grayness and baldness.

We often see a solution of borax, or soda, or hartshorn recommended as a wash for the hair. Such preparations can only result in injury to its texture.

### THE PEOPLE ARE STRONG.

Monopoly may boast its strength,
And it may rule and ruin long ;
In time its rope will reach its length,
Because the people still are strong,
And neither blind nor deaf nor dumb—
That time will come.

A selfish, greedy, grasping few
The many's temper still may try,
And do the worse that they can do,
To add, divide and multiply ;
But when their efforts reach their sum,
Their time will come.

Great is the patience of the free,
And they are often slow to move ;
But woe to those, whoe'er they be,
Who would too far that patience prove!
The day that strikes them stiff and numb
Will surely come.

### GROWS EITHER WET OR DRY.

Chief Justice Mansfield, probably with a view to prolong his own days, was always anxious, when old witnesses were in court, to know their customary habits of life. It so happened that two very old men named Elm were one day the objects of his inquiry.

"You are a very old man," said his lordship to the elder brother. "I suppose you have lived a very temperate life?"

"Never drank anything but water, my lord," said Elm.

"Nor you either, I suppose ?" said the judge, addressing himself to the younger.

"When I could get nothing else, my lord," was the reply. "I always took my glass with my friends."

"Well, then," replied his lordship, "all that we can say is 'an Elm will flourish wet or dry.'"

